# Engaging older adults in healthcare research and planning: a realist synthesis

**DOI:** 10.1186/s40900-016-0022-2

**Published:** 2016-03-07

**Authors:** Heather McNeil, Jacobi Elliott, Kelsey Huson, Jessica Ashbourne, George Heckman, Jennifer Walker, Paul Stolee

**Affiliations:** 1 School of Public Health and Health Systems, University of Waterloo, 200 University Avenue West, Waterloo, ON N2L 3G1 Canada; 2 Schlegel-UW Research Institute for Aging, Kitchener, ON Canada; 3 School of Human and Social Development, Nipissing University – Muskoka Campus, Bracebridge, ON Canada; 4 Conestoga College, School of Health & Life Sciences and Community Services, Waterloo, ON Canada

**Keywords:** Engagement, Research, Planning, Older adults, Realist synthesis

## Abstract

**Plain English summary:**

The importance of citizen involvement in healthcare research and planning has been widely recognized. There is however, a lack of understanding of how best to engage older adults, Canada's fastest growing segment of the population and biggest users of the healthcare system. We aimed to address this gap by developing an understanding of the engagement of older adults and their caregivers in healthcare research and planning. We conducted a review of available knowledge on engagement in healthcare research and planning with a focus on older adults and their caregivers. A five stage engagement framework emerged from this study that can be used to guide engagement efforts. We are continuing to collaborate with older adults and decision makers to develop and test strategies based on the presented framework.

**Abstract:**

**Background**

The importance of engaging the community in healthcare research and planning has been widely recognized. Currently however, there is a limited focus on older adults, Canada’s fastest growing segment of the population and biggest users of the healthcare system.

**Objective**

This project aimed to develop an understanding of engagement of older adults and their caregivers in healthcare research and planning.

**Method**

A realist synthesis was conducted of the available knowledge on engagement in healthcare research and planning. The search methodology was informed by a framework for realist syntheses following five phases, including consultations with older adults. The synthesis included theoretical frameworks, and both peer-reviewed and grey literature.

**Results**

The search generated 15,683 articles, with 562 focusing on healthcare research and planning. The review lead to the development of a framework to engage older adults and their caregivers in healthcare research and planning. The 5 stages *environment*, *plan*, *establish*, *build*, and *transition* are accompanied with example context, mechanism, and outcomes to guide the use of this framework.

**Conclusion**

We have identified a framework that promotes meaningful engagement of older adults and their caregivers. We are continuing to collaborate with our community partners to further develop and evaluate engagement strategies that align with the presented framework.

## Background

There is a growing realization of the importance of involving the public in the planning and development of public services [1]. This is increasingly relevant in healthcare, where it has been recognized that a critical unexploited resource for improvement is “the knowledge, wisdom and energy of individuals and families” (p.254) [2]. It has also been acknowledged that there is a disconnect between the literature on health interventions, and the degree of interest of patients [3]. In the current movement to support a shift from episodic models of healthcare to more integrated, people-centered ones, the engagement of those who are (or are at risk to be) high users of the healthcare system, as well as their families, is essential.

The rationale for engaging patients in healthcare research has been summarized well by a recent report commissioned by the United Kingdom National Health Service in that, “public involvement in research is founded on the core principle that people who are affected by research have a right to have a say in what and how research is undertaken” (p. 12) [4]. This democratization of research has lead to increased support for community engagement approaches in research circles for a variety of reasons, including the ability to reduce or eliminate vulnerable group health disparities [5]. Engagement is especially effective with hard-to-reach communities for whom data are limited or missing in pre-existing databases [6]. Older adults, an example of these communities, represent the largest growing segment of the population and greatest users of the healthcare system. Unfortunately, the current healthcare system has not been adequately designed to meet their needs [7]. In order to transform the system to support integrated care, the engagement of older adults in healthcare research and planning is essential.

It has been suggested that a specific strategy to achieve this engagement is collaboration. Trochim and Kane [8] acknowledge that complexities of the healthcare system require approaches to knowledge generation that encourage working across disciplines to include a diverse collaboration of stakeholders at all levels of the health system. For the purposes of this paper, this collaboration will be termed transdisciplinarity, a complex term which is described by Smith [9] as collaboration that provides a link between research knowledge and decision-making processes to seek solutions that are “feasible, socially acceptable, appropriate, effective and sustainable” (p.161). Given this recognition of the importance of working across stakeholder groups, the principles for engaging older adults and caregivers in healthcare research and planning should be examined together from a systems level.

For such an important concept, patient engagement is an ambiguous phrase. Terms like patient involvement, client engagement, public involvement, patient-centered care and others have been used synonymously in the literature to describe this idea. For the purposes of this paper, the term “engagement” will be used and defined as, “a relative term subjectively defined by individuals or groups/organizations that are planning to actively involve patients and their families in various health care advisory committees or care decision making” (p.4) [10]. This characterisation recognizes the importance of engaging families who play a significant role as care partners for older adults.

Despite interest in engagement in healthcare research and planning, there has not been, to our knowledge, a systematic review to understand the principles underlying engagement of older adults. A recent review by Domecq and colleagues [11] studied patient engagement in research in the general population and concluded that while engagement is feasible, more research is needed to understand the best methods to actualize this engagement. Similarly, Staniszewska and colleagues [12] have suggested that “patient-based evidence” is an important and lacking component of healthcare research. Our study builds knowledge in this negated and necessary area to improve integrated care by asking the question: what are the underlying principles needed to operationalize engagement of older adults in healthcare research and planning? Specifically this realist synthesis addresses i) the contextual factors that influence meaningful engagement of older adults in healthcare research and planning; ii) the outcome (levels of engagement) achieved through various engagement opportunities; and ii) the mechanisms necessary to achieve meaningful engagement in healthcare research and planning.

## Review

### Methodology

#### Study design – the realist synthesis method

A type of synthesis that allows for considerations of what engagement approaches may work for older adults and the contextual influences [13, 14] to consider was necessary to answer this project’s research questions. Realist syntheses are a type of scoping review methodology developed by Pawson [15] and Greenhalgh [16] and their colleagues. Major research funding agencies have recognized these methods as appropriate knowledge synthesis approaches [17] because they address limitations of more traditional systematic reviews and meta-analyses. These limitations include: narrowly defined effectiveness and a lack of explanation of the circumstances in which an intervention or policy does or does not work. Realist syntheses provide the necessary richness of information and explanation to guide real-world decision-making. They also evaluate a broad range of empirical evidence and assess value by allowing for an understanding of the contribution of a work rather than according to some preset criteria [13]. Kastner and colleagues [18] recognize the value of the realist synthesis when reviewing mixed types of evidence. In undertaking this synthesis, peer-reviewed and grey literature were appraised; conceptual/theoretical as well as empirical work was analyzed; research conducted using qualitative, quantitative and mixed methods and expert opinion (including the perspectives of older adults and their caregivers) was gathered.

The realist synthesis is a relatively novel and increasingly popular form of scoping review whose best practice standards are still in consideration [14]. As such, decisions were made early on in the project to follow Wong and colleague’s [14] framework for realist syntheses. The realist synthesis processes of scope clarification, stakeholder involvement, systematic search and review and development/ dissemination of recommendations are consistent with accepted methods of best practice guideline development.

The five phases of the realist synthesis are similar to the steps undertaken in a traditional Cochrane review but are iterative and overlapping [15], reflecting the real-world application of the information being synthesized. More details on the methods for this study can be found in a published protocol [19].

#### The five phases of realist synthesis

***Phase One: Clarifying Scope:*** In order to satisfy the realist synthesis goal of refining the review question and identifying a candidate theory to populate [15], four sub-phases were conducted: initial consultations with stakeholders, a grey literature search, key informant interviews and a workshop.

Eight members of the SHARP (*Seniors Helping as Research Partners)* network were consulted to consider the project goals and the meaning of engagement. SHARP began in June 2013 as an effort to meaningfully engage older adults in the work of our research group from the partnerships and collaborations we have developed within the community, and the feedback received from these community members on their desire to be more involved in healthcare-related research. We have now recruited over 70 older adults to be engaged to their desired level in our research and planning efforts.

This consultation was followed by a grey literature search to identify candidate theories, which was conducted with snowballing techniques on Google. There was an initial focus on Canadian information at the provincial and national levels, but frameworks used internationally were identified through hand searching from countries with known interest in engagement (e.g. The United Kingdom). Two 30-minute key informant interviews with research leaders recognized as experts in healthcare engagement in Canada were conducted, audio-recorded and later transcribed. Data were coded using Lofland and colleagues [20] line-by-line coding technique.

The final step to clarify the scope was a full-day workshop conducted with 17 participants from Patients Canada, “a patient-led organization that fosters collaboration among patients, family caregivers and the healthcare community”, who discussed the meaning of patient engagement and reviewed the frameworks identified from the previous sub-phases. Note-takers recorded information that was later analyzed. During this phase the “program theories” emerged (p.1) [16] to be used throughout the review.

The components of this project involving community consultation received ethics clearance from the University Of Waterloo Office Of Research Ethics (ORE# 19094).

***Phase Two: Search for Evidence:*** The second phase of this project involved an extensive, purposive search of the peer-reviewed literature. **Search Methodology:** A systematic search of the following licensed databases was conducted: MEDLINE, EMBASE, CINAHL, Sociological Abstracts, Scopus and the Cochrane Database of Systematic Reviews from the earliest coverage of these databases to the date of the final search, January 2014. A description of the search terms used is described in the protocol for this review [19] including synonymous terms for engagement and healthcare research and planning. The search results were exported to RefWorks, a reference management system and duplicate results were deleted. **Inclusion and Exclusion Criteria:** Papers were included if they reported a description, assessment or evaluation of strategies for engagement of adults (18+), families or caregivers. Papers containing strategies relevant to older adults (age 65+) were highlighted in the abstraction. Papers focusing strictly on engagement of individuals under the age of eighteen were excluded. The review included both English and French language content.

***Phase Three: Appraise primary studies and extract data:*** Understanding that in realist syntheses, data abstraction is an on-going, iterative process, abstraction guidelines outlined by Pawson and colleagues [15] and by Wong and colleagues [14] were followed. The abstraction table was developed in consultation with older adults to extract the necessary data. In the appraisal of the studies, questions influenced by Kastner and colleagues [21] were asked to assess relevance. Rigour, or the quality of the article was assessed [14].

***Phase Four: Synthesize evidence and draw conclusion:*** Using an emergent approach, article abstraction to saturation and line-by-line coding were conducted independently by the investigators [20]. Random sampling was employed to select articles for coding. As each article was reviewed, and re-read, codes to capture themes or concepts related to the initial rough theories and engagement opportunities that emerged from the data were created and iteratively revised. For a realist synthesis, Pawson and colleagues [22] suggest reviewers should aim for theoretical saturation rather then encyclopaedic coverage. Through an iterative process, the emerging themes were used to populate the candidate program theories that both confirmed and refined components of the frameworks as described in the methodology of Wong and colleagues [14]. Through frequent research team meetings the data were examined for information related to the context, potential mechanisms and outcomes (CMOs) of meaningful engagement. Central to realist syntheses, these CMO structures aim to explain, within a particular context, what underlying process (mechanism) occurs to achieve a specific outcome [14]. CMOs are useful in supporting or refining the original theories to develop the final program theory displayed in the results section.

***Phase Five: Disseminate, implement and evaluate:*** A half-day workshop with 11 participants from Patients Canada was conducted to discuss the findings from the synthesis; notes from the discussions were recorded by three students. The information gathered from this consultation session was synthesized to finalize the principles for engagement of older adults in healthcare research and planning.

### Results

***Phase One:*** Initial consultations with the SHARP network helped to shape an understanding of the term “engagement” from the perspective of older adults and to confirm the validity of the research question.

Further clarification came from the key informants. They suggested one commonly used framework in the area of patient and citizen engagement, the *Spectrum of Engagement* [23]. This framework describes engagement as ranging from an “inform” level where the public is provided with information, to increasing levels of public engagement with “empower” at the highest end, where final decision-making is in the hands of the public. Key informants both emphasized the role that the context plays in engagement opportunities,

*“it’s not that you can take the framework and apply it across the whole spectrum of care for that particular group, it’s based on that particular situation that is impacting them for the moment, and it might be a very specific kind of approach or strategy, very specific…”* (Key Informant*,* personal communication, 2014)*.*

The information from these two sub-phases was helpful in shaping the grey literature search that retrieved a number of frameworks. These were abstracted and discussed, highlighting eight frameworks for further review: Consumer, Carer and Community Engagement Model [24]; Shared Decision Making Model [25]; Person-Centered Practice Conceptual Framework [25]; Ladder of Citizen Participation [26]; Spectrum of Engagement [23], Spectrum of Participation [27]; Community Engagement Model [28]; and the 8 Dimensions of Patient-Centered Care [29].

After reviewing each framework with members of Patients Canada (as described above) the participants suggested two frameworks that could be used to guide the review. The Spectrum of Participation [27] presents the same stages of engagement as the Spectrum of Engagement, but has evolved into a circle. This is an important difference for older adults as participants perceive the circle as more of a depiction of reality, highlighting that people should be able to move between any level, at any period of time. One participant said,

*“I can understand this model [Spectrum of Participation], which is important. For a diagram to work, it should be intuitively comprehensible”* (Participant, personal communication, 2014).

The 8 Dimensions of Patient-Centered Care [29] was also selected as representing areas that would be necessary to consider in engaging older adults in healthcare research and planning.

**Fig. 1:**
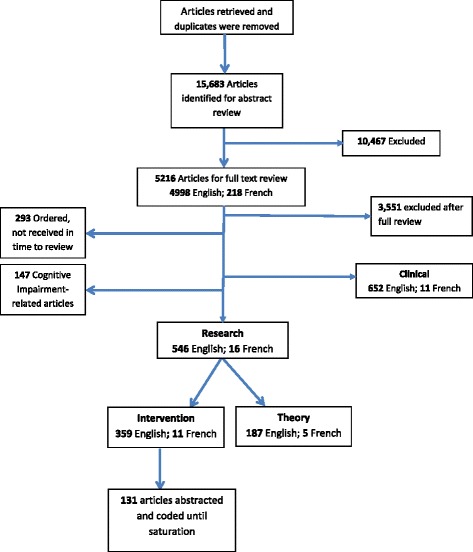
Peer reviewed literature sorting

***Phase Two and Three:*** Fig. 1 shows the number of studies included at each stage of the review. The search yielded a total of 15, 683 articles once duplications were removed. The articles first underwent a title and abstract review; 10,467 articles were excluded. Six hundred and fifty-two articles focused on engagement in healthcare decision-making, these were set aside for another realist review conducted by the *GHS Research Group.* One hundred and forty-seven articles that focused on cognitive impairment were also set aside as engagement techniques for this population might be unique and require further analysis. The remaining 562 articles (546 English research/ planning papers and 16 French) underwent a full-text review for this manuscript. Reviewers independently sorted articles into three categories; Exclude, Theory or Evidence/Intervention, one reviewer was responsible for the French language articles. The criteria for inclusion were met if the article reported a description, assessment or evaluation of strategies for engagement of adults (18 years and older), families or caregivers. Articles were excluded if the article focused on individuals under the age of 18. Those that focused on theoretical development were excluded from the review but were examined for relevant models and were retained to inform the discussion of this paper.

To assess inter-reviewer agreement on article retention, a sample of articles was reviewed independently by two reviewers and results were compared using a kappa statistic [17] where a score of *k = 0.63* was achieved, considered “good” using Altman’s [30] criteria (>.60). The reviewers met numerous times throughout the sorting process to discuss and remain consistent.

Of the 546 English articles, 187 articles focused on theory (meaning that they did not discuss a specific engagement experience or initiative) and were set aside to inform discussion and theory development, the remaining 359 were considered to have sufficient evidence to be included in the final sample for data abstraction. Of the 16 French studies, five focused on theory and 11 were included in the final sample to be abstracted. In total, the final sample of included articles was 370. One hundred and thirty one of these were abstracted and coded before the reviewers reached theoretical saturation.

***Phase four:*** Analysis of the articles revealed that the majority of the work in this area was conducted in the United Kingdom (33 studies, 25 %). Fifteen (11 %) were conducted in Canada, 8 (6 %) in the United States, 5 (4 %) had a multinational focus.

There was a mix of articles focused on research and planning; 71 (54 %) of the articles focused on engagement in research, while 60 (46 %) discussed planning. Sixty-six percent (86) of the articles discussed engagement strategies that could be described as *Involvement* (e.g., system involves stakeholders in planning and policy processes through workshops) according to the Spectrum of Participation [27] while 20 % (26) of the articles were at the level of *Empowerment* (e.g., community identifies issues and solutions).

In this phase, participant characteristics in terms of identified sub groups were analyzed. The majority of the articles (77 studies, 59 %) did not discuss a specific participant group. An example of an identified group that was discussed can be broadly termed aboriginal peoples from various nations. While this subgroup was represented in six articles (5 %), there were no significant differences in the engagement strategies discussed or the barriers/ facilitators to involving this population; the strategies mentioned were similar to those in the broader population [31, 32].

Eleven (8 %) of the articles focussed on engaging older adults. When these articles were compared to the others in the sample, there were no significant differences related to engagement, except that age was discussed as an important consideration, where a participants’ age related characteristics were found to play a role in the opportunity for meaningful engagement, for example in the accessibility of engagement opportunities [33–35]. Despite the relatively small sample of articles focused specifically on engaging older adults, a central outcome of the next phase discussed below was the validation of our framework through consultations with older adults and their caregivers. The consultation process provided us with confidence in the usefulness and appropriateness of our framework for this population and suggests a gap in the research that we have helped to address.

***Phase five:*** Analysis of the literature has identified a framework for engaging older adults and caregivers in healthcare research and planning. Central to these is the development of a relationship between all stakeholders involved based on communication and an understanding of context-specific desired levels of engagement.

**Fig. 2:**
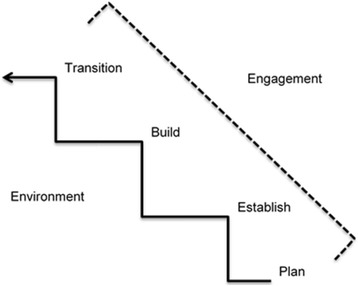
Program theory: Framework for engaging older adults in healthcare research and planning

The participants at the half-day workshop held with Patients Canada felt that the 8 Dimensions of Patient-Centered Care [29] was no longer relevant to understanding engagement in healthcare research and planning. One participant told us, “*this just doesn’t make any sense for research*” (Participant, personal communication, 2014). The investigators had discussed challenges with this framework during the abstraction phase of the project and agreed with the suggestion of the workshop participants to retain elements of the framework that were appropriate, such as ensuring an understanding of participant (patient) preferences, but to continue to search for evidence that moves beyond this framework. In the analysis that followed, elements which emerged from the literature and consultations helped to develop the following program theory (discussed throughout as a “framework” for clarity to the reader) which can be used to guide meaningful engagement with older adults and their caregivers in healthcare research and planning (Fig. 2).

#### Environment

Considerations at a system level, which we termed *environment* to capture the breadth of this contextual influence, impact engagement opportunities for older adults and their caregivers. This occurs in complex ways, ranging from the impacts of the physical environment and resources, to policy. Policy guides engagement initiatives [36, 37] that influence organizational support. This support from the “top” is central to the success of engagement opportunities in order to address issues surrounding power imbalances between stakeholders [38, 39]. In this way, the environment may affect meaningful engagement opportunities for older adults and their caregivers.

At a more direct level, issues such as accessibility need to be taken into consideration for engagement opportunities to be successful. Disability associated with a mismatch between age related challenges and the current environment affect the opportunity to meaningfully engage older adults who represent a spectrum of ability levels. Mobility issues are an example of this. The physical location of engagement opportunities plays a role in the participation of older adults. The building where participants are being engaged needs to be accessible [33, 34] to limit barriers facing older adults who experience age-related changes (e.g., functional decline). The scheduling of engagement opportunities should ensure that older adults are able to participate [35]; transportation is a related area of consideration [40]. Other indirect factors that affect engagement include training, scheduling, health system models, practices and culture.

There is a need to understand participant motivation to participate in research and planning. Remuneration should be examined to compensate participants for their contribution to research and planning [35, 41, 42] because of the time investment required for meaningful engagement [43–45].

#### Plan

Once the environmental context is understood, it is important to plan for meaningful engagement of older adults and their caregivers in healthcare research and planning. To do this, the literature suggests that the stakeholders involved must have a mutual understanding of each other.

Investigators need to understand the characteristics [46, 47], demographics [33, 38], preferences [48, 49], goals [50, 51], expectations [52, 53] and needs [35, 43] of those with whom they are engaging. Skills [54, 55] and knowledge [56, 57] of participants also influence how older adults engage in healthcare research and planning. It is important for researchers and decision-makers to tailor engagement approaches so that they are appropriate for their target group [58].

The participant group should be composed of a diverse and representative sample of the population in question [49, 59]. Part of this diversity is the different preferences participants have for engagement. Investigators need to create opportunities for participants to be engaged in research and planning at the level of their choice [38, 60]. Once this level is mutually understood, the engagement of the social support network (family, friends, peers and neighbours) should be considered, as they can provide valuable insight when researching and planning healthcare for older adults [38, 61].

In the planning stages it is also important to understand who is conducting research and making decisions in the healthcare system. Through group discussion the authors decided that “Investigators” was the best term to use when discussing researchers and decision-makers involved in engaging older adults. The literature supports the relationship between investigator characteristics [50, 52, 62, 63], preferences [64], goals [50, 65], expectations [35, 39, 60] and needs [64, 65] and their engagement with older adults. Investigator attitudes [55, 66], skills [57, 58] and knowledge [65, 67] affect engagement in healthcare research and planning. For example, collaboration is a skill that is often unfamiliar to researchers [68] but important to engagement. Investigators must be reflective [69] in order to recognize their skill levels and seek training when necessary.

#### Establish

Relationships are central to the engagement of older adults and caregivers in healthcare research and planning. This stage of engagement is important regardless of the length, type or intensity of the engagement opportunity.

In order to establish relationships among stakeholders involved in healthcare research and planning, trust [43, 70], role clarity [71, 72], respect [33, 67, 73], communication [56, 74] and information sharing [46, 48] are necessary. Other commonly cited factors that can encourage relationship building include i) flexibility, both in methodology/ structure and stakeholder openness to change [33, 49, 50], and ii) efforts to deformalize engagement opportunities and encourage more casual and open conversation (e.g. the provision of food) [33, 35, 51, 67].

Negative views and mistrust with healthcare and research [40] are barriers discussed both in the literature and in participant consultation, preventing meaningful engagement at all levels of participation in healthcare research and planning. One step investigators can take to overcome these barriers is asking participants about their preferred level of engagement [68] and planning accordingly.

These seemingly straightforward recommendations must be taken seriously, as the time needed to develop meaningful relationships is often not available [40, 49]. A culture of engagement needs to be encouraged in research institutions and the healthcare planning sphere. Education of all stakeholders on the benefits of engagement could be a step towards the necessary culture shift [75].

#### Build

Sometimes the goal of the engagement opportunity is to build an ongoing, sustainable relationship between stakeholders. In such cases, a co-production approach is suggested between stakeholders involved in healthcare research or planning. Though not always a stage of meaningful engagement, stakeholders can build on an established relationship by co-producing the processes and desired outcomes of healthcare research and planning.

Research and planning methods influence engagement opportunities for older adults and their caregivers and are important to examine when building a long-term relationship. Although engagement is more commonplace in qualitative or participatory research methods [4] it can be incorporated into research and planning [54, 76] even in traditionally non-participatory methods, such as randomized controlled trials [77]. Investigators can engage with the public (e.g., in an advisory capacity) from the beginning of the project, involving older adults in deciding on the research question. Investigators can effectively make use of partnerships with communities by developing mutually agreed upon research agendas, timelines and goals [78].

Efforts should be made to apply and evaluate all strategies for engagement periodically [79]. Engagement in healthcare research and planning might vary throughout a project, depending on the chosen methodology, skills and knowledge of participants, and their desire to be involved. Discussing the expected outcome of engagement as well as outcomes of research and planning with older adults and their caregivers is an opportunity within a long-term relationship to encourage meaningful engagement.

#### Transition

For many engagement opportunities a transition of engagement opportunities and relationships is realistic. For example, if a project is coming to an end, decisions about how to conclude or modify a relationship are important. Knowledge translation is a key mechanism for the continued development of participant and investigator engagement through the inevitable process of transition. Workshop participants shared with us that disseminating research results encourages continued engagement through the development of a meaningful relationship. The provision of timely feedback to participants is important in encouraging engagement [80]. Continuity of stakeholders and their efforts to engage were also found to be significant throughout the process of information exchange [34, 41, 81]. Knowledge translation plans should be created in collaboration with older adults and caregivers as it is essential to have ongoing collaborations with knowledge users who represent the community of interest.

The format of information is vital; cultural appropriateness and literacy levels should be assessed in the creation and dissemination of information to older adults and caregivers.. The amount of information provided to participants needs to be considered, as a lack of information reduces engagement opportunities [82]. Information provided to older adults and their caregivers should be unbiased [83] and accessible in terms of language, cultural preferences, education level and age associated decline [31, 58].

#### CMO examples

To review the framework and gain insight from older adults and their caregivers into how to understand the context and mechanisms necessary to achieve outcomes of meaningful engagement in healthcare research and planning, we again engaged our stakeholders. Example CMOs are presented to display specific contexts, underlying mechanisms and subsequent outcomes that can be used to providing direction for understanding the “how”, “why” and “in what context” meaningful engagement of older adults and their caregivers occurs while implementing our framework. These CMOs are useful in understanding the framework stages as stepwise, beginning with developing an understanding of the environment, following to planning the engagement opportunity, establishing and building a relationship and transitioning. Readers should note that the build stage should be considered but might not always be necessary depending on the goals of the engagement opportunity.

#### Environment

The first stage in meaningful engagement of older adults in healthcare research and planning is to develop an understanding of the underlying context of the environment (including funding opportunities and arrangements, system constraints and the physical environment). For example, in the *context* of a collaborative research environment, the *mechanism* of mutual valuing of experiences and knowledge will lead to the foundations necessary for the *outcome* of planning an engagement opportunity.

Since the context of the environment is complex and dynamic, it is important to monitor this throughout engagement opportunities in healthcare research and planning. Once the underlying environmental context is understood, the following four stages of engagement are useful in establishing meaningful engagement with older adults.

#### Plan

In the context of planning an opportunity for meaningful engagement of older adults in healthcare research or planning, it is important for stakeholders to be aware and develop an understanding of the values and norms of the differing groups. This should include a conversation about timelines and goals of the engagement opportunity; the Spectrum of Participation [27] is useful in understanding desired levels of engagement. The outcome of trust will be generated, identified in the literature as an important component of meaningful engagement.

#### Establish

Once the engagement opportunity has been planned, the context of establishing an engagement relationship follows. The mechanism here is the resource of time. By investing time in working together, the outcome is that each group (older adults and investigators) begin to develop respect for the other, creating the relational foundation on which meaningful engagement can be built. From here engagement at all levels of the Spectrum of Participation [27] can take place, regardless of time, commitment or type of project.

#### Build

Once a relationship is built, it is possible to sustain partnerships in healthcare research and planning through the mechanism of flexibility (in terms of time commitments, deadlines and schedules). An outcome of ongoing investment of all stakeholders in the partnership will be achieved. If a partnership is not the goal of the engagement opportunity, this stage might not be a part of the engagement opportunity.

#### Transition

In the context of transitioning relationships in healthcare research and planning, it is important for stakeholders to learn from each other through the mechanism of co-production to achieve the outcome of older adults feeling valued.

Knowledge translation is a useful tool for sustained feelings of value in transition and beyond the course of an engagement opportunity. Through the co-production and translation of knowledge, older adults and their caregivers have the opportunity to learn from the research or planning process and feel valued. This important component of meaningful engagement is useful in overcoming the common criticism of tokenistic engagement in healthcare research and planning efforts.

### Discussion

This review has contributed to the underdeveloped evidence of older adult engagement in healthcare research and planning. We have outlined a program theory and associated CMOs to operationalize engagement of older adults in this area. The results of this synthesis reveal that engagement strategies for the general population are relevant to older adults and caregivers. Significantly, because of our consideration of the caregiver, this realist synthesis has unearthed a framework that applies to engagement involving any (adult) age group. Our approach to this topic from the specific direction of age serves to highlight relevant aspects of meaningful engagement for older adults and caregivers. We therefore suggest that engagement opportunites be understood within the specific environment in which they are being implemented using the framework presented to understand the context, mechanisms and outcomes of meaningful engagement in healthcare research and planning.

Engagement of older adults and their caregivers in healthcare research and planning needs to be understood as a relationship in which considerations of the participants, investigators and the environment are dynamic and responsive to each other. The presented engagement framework aligns well with frameworks in this field that suggest the importance of partnership [84, 85]. Engagement of older adults and their caregivers in healthcare research and planning is a step towards an integrated system in which the patient perspective is appreciated. Ferrer and Goodwin [86] have discussed the importance of involving “people and communities as co-producers of care” (p.1) as a component of integrated care, citing “co-production” as a core principle guiding people-centered and integrated health services. The results of this project align with this notion and present a framework that could be implemented to achieve this.

The work of Carman et al. [87] incorporates certain similar aspects to our framework, recognizing the importance of partnership between all stakeholders involved in engagement. The development of our framework however engaged older adults throughout the entire process, generating important insights, which are reflected in our framework. For example, older adults involved in our project did not like the hierarchical structures that dominate the commonly cited frameworks of engagement.

The engagement framework that has developed out of this synthesis supports an evolution of the definition of patient engagement. The definition offered in the introduction of this paper by Gallivan and colleagues [10] presents participants in engagement as passive observers, suggesting that engagement is something that the organization/ investigator allows to happen. From this review we understand that true engagement breaks down power differentials and creates partnerships that are meaningful to all stakeholders involved. Specifically, to support the presented framework of older adult and caregiver engagement in healthcare research and planning, a revised engagement definition needs to discuss the idea of relationship based on authenticity, communication and expectations. The work of Supple [88] and colleagues aligns with this understanding, presenting principles aligned with meaningful engagement including involving patients early and thoughtfully in projects and throughout the dissemination process.

In regards to authenticity, participants want to know that their engagement in research will actually have an impact on their experience of the healthcare system. Older adults want to see the “fruits” of their engagement; their input affecting the processes and planning of the healthcare system. In order for this to happen, trust needs to be built. The importance of trust has been widely studied [89, 90] and linked to discussions between stakeholders about the outcomes and goals of the research or planning projects being conducted at the beginning of a project. In doing so, expectations can be understood early on and will be more likely met at the conclusion of a project.

It is essential to think about the level of engagement of older adults and their caregivers in healthcare research and planning. Some participants want to be, and are capable of being, involved in research from the beginning of the project, for example, in the formation of research questions. Others are content to participate at more of an “inform” level [27]. It is vital that investigators collaborate with the community early on in the research cycle to understand this desired level of engagement and plan accordingly. If a relationship is fostered between stakeholders, older adults will be more likely to view their role in research and planning as empowering. To understand this desired level, communication is necessary. Communication has been identified as a critical component for integrated healthcare [91, 92]. This project extends the significance of this concept to engagement of older adults in healthcare research and planning. Avoidance of tokenism is critical to encourage older adult engagement in research. As highlighted by others in the field, expectations and past experiences are critical factors influencing participants’ willingness to engage in research [34, 93]. This project expands on this knowledge to understand that if a researcher promises engagement at a level of empowerment only to provide opportunities that are at a less influential level (e.g. information provision), older adults and their caregivers will likely become disenfranchised from wanting to be engaged in future healthcare research and planning opportunites.

#### Strengths and limitations

By using a realist synthesis method, the review has begun to explain, “how”, “why” and “in what context” engagement occurs. A realist synthesis is subjective and interpretive in nature, and although our methods and steps have been documented, other researchers reviewing the same literature may arrive at different conclusions [14]. An example of this is the selection of our program theories. Throughout this process it has been recognized that there are many other theories and frameworks for engagement, however information from our key informants and the focus of our search directed us to the candidate theories chosen for this review. Participants in our consultation methods provided feedback on the theories, a process that is unique to a realist synthesis. This feedback provides support for the theories chosen.

Because of the breadth of knowledge available on patient engagement, a seemingly unmanageable number of articles were generated through our search. Our methodology, such as the implementation of random sampling of the articles for abstraction and the use of theoretical saturation, resolved this issue to provide a range of studies creating a robust basis for the development of the presented framework.

Despite the abundance of literature on engagement, there were a limited number of articles that focused on engaging older adults. The realist synthesis methodology was beneficial in remedying this, as the consultations held with older adults in the community contribute to, and verify, the knowledge gained through the literature review. Despite our best efforts, there was a lack of diversity in the older adults with whom we consulted with; the participants were mainly Caucasian. In future projects, we plan to follow the recommendations from this review and recruit a more diverse sample by developing relationships with minority communities, reaching out to stakeholders and broadening our existing networks and once contacted working to develop trust in a co-production approach to understanding their motivations to participate.

Another lesson that was learned in this review came by way of self-reflection and an understanding of the presented framework. Although older adults and their caregivers were involved from the conception of this project, their expectations and preferred level of engagement was not discussed with them from the inception of the project. Engaging older adults in a meaningful relationship will take time. We have begun to build this relationship and will continue to follow the proposed framework.

## Conclusions

Engagement of older adults and their caregivers in healthcare research and planning is complex and should be viewed as a dynamic relationship between stakeholders. The engagement of older adults and caregivers in healthcare research and planning should be authentic, appropriate for their desired level of engagement and understood within the context of the environment. Communication to develop a relationship or partnership (if that is the goal of the engagement) is central to engaging older adults in healthcare research and planning.

Results from this project can be used to support the meaningful engagement of older adults and caregivers in research and planning necessary to move towards integrated healthcare. Engagement of those whom research and planning will most affect will better guide health system priorities and create an evidence base that can inform priorities for policy and healthcare system planning. The next steps of this project include continued collaboration with our community partners to evaluate the framework presented in this manuscript so that they can be used in the development and evaluation of healthcare research and planning engagement toolkits.

## References

[1] Holosko MJ, Leslie DR, Cassano DR (2001). How service users become empowered in human service organizations: the empowerment model. Int J Health Care Qual Assur.

[2] Doherty W, Mendenhall T (2006). Citizen health care: A model for engaging patients, families, and communities as coproducers of health. Fam Syst Health.

[3] Tallon D, Chard J, Dieppe P (2000). Relation between agendas of the research community and the research consumer. Lancet.

[4] Staley K (2009). Summary Exploring Impact: Public Involvement in NHS, Public Health and Social Care Research.

[5] Morone JA, Kilbret EH (2003). Power to the people? Restoring citizen participation. J Health Polit Policy Law.

[6] Nguyen G, Hsu L, Kue K, Nguyen T (2010). Partnering to collect health services and public health data in hard-to-reach communities: A community-based participatory research approach for collecting community health data. Prog Community Health Partnersh.

[7] Heckman GA (2011). Integrated care for the frail elderly. Healthc Pap.

[8] Trochim W, Kane M (2005). Concept mapping an introduction to structured conceptualization in health care. Int J Qual Health Care.

[9] Smith PM (2007). A transdicisplinar appracoh to research on work and health: what is it, what could it contribute, and what are the challenges?. Crit Public Health.

[10] Gallivan J, Kovacs Burns K, Bellows M, Elgenseher C (2012). The many faces of patient engagement. J Particip Med.

[11] Domecq JP, Prutsky G, Elraiyah T, Wang Z, Nabhan M, Shippee N (2014). Patient engagement in research: a systematic review. BMC Health Serv Res.

[12] Staniszewska S, Crowe S, Badenoch D, Edwards C, Savage J, Norman W (2010). The PRIME project: developing a patient evidence-base. Health Expect.

[13] Gough D, Thomas J, Oliver S (2012). Clarifying differences between review designs and methods. Syst Rev.

[14] Wong G, Greenhalgh T, Westhorp G, Buckingham J, Pawson R (2013). RAMESES publication standards: realist syntheses. BMC Med.

[15] Pawson R, Greenhalgh T, Harvey G, Walshe K (2005). Realist review- a new method of systematic review designed for complex policy interventions. J Health ServResPolicy.

[16] Greenhalgh T, Wong G, Westhorp G, Pawson R (2011). Protocol-realist and meta-narrative evidence synthesis: Evolving standards (RAMESES). BMC Med Res Methodol.

[17] Graham I. Knowledge synthesis and the Canadian Institutes of Health Research. Syst Rev. 2012;1(6). doi: 10.1186/2046-4053-1-6. 10.1186/2046-4053-1-6 PMC3351743 22587985

[18] Kastner M, Tricco A, Soobiah C, Lillie E, Perrier L, Horsley T (2012). What is the most appropriate knowledge synthesis method to conduct a review? Protocol for a scoping review. BMC Med Res Methodol.

[19] Stolee P, Elliott J, McNeil H, Boscart V, Heckman GA, Hutchinson R (2015). Choosing healthcare options by involving Canada’s Elderly: a protocol for the CHOICE realist synthesis project on engaging older persons in healthcare decision-making. BMJ Open.

[20] Lofland J, Snow DA, Anderson L, Lofland LH (2006). Analyzing social settings: a guide to qualitative observation and analysis.

[21] Kastner M, Estey E, Parrier L, Graham I, Grimshaw J, Straus S (2011). Understanding the relationship between the perceived characteristics of clinical practice guidelines and their uptake: protocol for a realist review. Implement Sci.

[22] Pawson R, Greenhalgh T, Harvey G, Walshe K (2004). Realist synthesis: an introduction. ESRC Research Methods Programme.

[23] International Association for Public Participation (IAP2). Spectrum of engagement. 2007. http://iap2canada.ca/Resources/Documents/IAP2%20Spectrum_vertical.pdf. Accessed 12 Mar 2014.

[24] National Consumer and Carer Forum of Australia. Consumer, Carer and Community Engagement Model. 2004. http://www.health.wa.gov.au/hrit/docs/publications/WA_Health_Consumer_Apr07.pdf. Accessed 16 Apr 2014.

[25] Government of New Brunswick. Primary Health Care Framework for New Brunswick. 2013. http://www.gnb.ca/0053/phc/pdf/2012/8752_EN%20Web.pdf. Accessed 30 Apr 2014.

[26] Arnstein SR (1969). A ladder of citizen participation. JAIP.

[27] Vancouver Coastal Health. Community Engagement Framework: Spectrum of Participation. 2009. http://www.vch.ca/media/CE%20Booklet%202009.pdf. Accessed 3 May 2014.

[28] Manitoba Family Services and Housing. Community Engagement Model. 2008. http://www.gov.mb.ca/fs/ce/pubs/community_engagement_framework_May08.pdf. Accessed 14 Mar 2014.

[29] National Research Corporation. 8 Dimensions of Patient-Centred Care. 2014. http://www.nationalresearch.com/products-and-solutions/patient-and-family-experience/eight-dimensions-of-patient-centered-care/. Accessed 5 Mar 2014.

[30] Altman DG (1991). Practical statistics for medical research.

[31] Maar MA, Lightfoot NE, Sutherland ME, Strasser RP, Wilson KJ, Lidstone-Jones CM (2011). Thinking outside the box: Aboriginal people’s suggestions for conducting health studies with aboriginal communities. Public Health.

[32] Wesche S, Schuster RC, Tobin P, Dickson C, Matthiessen D, Graupe S (2011). Community-based health research led by the Vuntut Gwitchin First Nation. Int J Circumpolar Health.

[33] Abelsohn KA, Ferne JM, Scanlon KA, Giambrone BL, Bomze SB (2012). ‘About time!’ insights from research with pride: A community student collaboration. Health Promot Int.

[34] de Wit MPT, Berlo SE, Aanerud GJ, Aletaha D, Bijlsma JW, Croucher L (2011). European League Against Rheumatism recommendations for the inclusion of patient representatives in scientific projects. Ann Rheum Dis.

[35] Delgado M (1996). Aging research and the Puerto Rican community: The use of an elder advisory committee of intended respondents. Gerontologist.

[36] Israel BA, Coombe CM, Cheezum RR, Schulz AJ, McGranaghan RJ, Lichtenstein R (2010). Community-based participatory research: A capacity-building approach for policy advocacy aimed at eliminating health disparities. Am J Public Health.

[37] Meier BM, Pardue C, London L (2012). Implementing community participation through legislative reform: A study of the policy framework for community participation in the western cape province of south Africa. BMC Int Health Hum Rights.

[38] Daykin N, Sanidas M, Tritter J, Rimmer J, Evans S (2004). Developing user involvement in a UK cancer network: Professionals’ and users’ perspectives. Crit Public Health.

[39] Alborz A, Wilkin D, Smith K (2002). Are primary care groups and trusts consulting local communities?. Health Soc Care Community.

[40] McKee M, Thew D, Starr M, Kushalnagar P, Reid JT, Graybill P (2012). Engaging the deaf American Sign Language community: Lessons from a community-based participatory research center. Prog Community Health Partnersh.

[41] Boote J, Baird W, Sutton A (2011). Public involvement in the systematic review process in health and social care: A narrative review of case examples. Health Policy.

[42] Royle J, Oliver S (2004). Consumer involvement in the health technology assessment program. Int J Technol Assess Health Care.

[43] Lantz PM, Viruell-Fuentes E, Israel BA, Softley D, Guzman R (2001). Can communities and academia work together on public health research? Evaluation results from a community-based participatory research partnership in Detroit. J Urban Health.

[44] Graham K, Chandler-Coutts M (2000). Community action research: Who does what to whom and why? Lessons learned from local prevention efforts (international experiences). Subst Use Misuse.

[45] Lenaghan J (1999). Involving the public in rationing decisions, the experience of citizens juries. Health Policy.

[46] Teunissen T, Visse M, de Boer P, Abma TA (2013). Patient issues in health research and quality of care: An inventory and data synthesis. Health Expect.

[47] White GW, Nary DE, Froehlich AK (2001). Consumers as collaborators in research and action. J Prev Interv Community.

[48] Chung P, Grogan CM, Mosley JE (2012). Residents’ perceptions of effective community representation in local health decision-making. Soc Sci Med.

[49] Sadler LS, Newlin KH, Johnson-Spruill I, Jenkins C (2011). Beyond the medical model: Interdisciplinary programs of community-engaged health research. Clin Transl Sci.

[50] Andrews JO, Newman SD, Meadows O, Cox MJ, Bunting S (2012). Partnership readiness for community-based participatory research. Health Educ Res.

[51] Tedford Gold SK, Abelson J, Charles CA (2005). From rhetoric to reality: Including patient voices in supportive cancer care planning. Health Exp.

[52] Boivin A, Currie K, Fervers B, Gracia J, James M, Marshall C (2010). Patient and public involvement in clinical guidelines: International experiences and future perspectives. Qual Saf Health Care.

[53] Warburton J, Bartlett H, Carroll M, Kendig H (2009). Involving older people in community-based research: Developing a guiding framework for researchers and community organizations. Australas J Ageing.

[54] O'Hagan B, Squire S, Powell C (2005). Five steps for sustaining effective patient partnership working. Nurs Times.

[55] Oliver S, Clarke-Jones L, Rees R, Milne R, Buchanan P, Gabbay J (2004). Involving consumers in research and development agenda setting for the NHS: Developing an evidence-based approach. Health Technol Assess.

[56] Slade M, Bird V, Chandler R, Fox J, Larsen J, Tew J (2010). The contribution of advisory committees and public involvement to large studies: Case study. BMC Health Serv Res.

[57] Telford R, Boote JD, Cooper CL (2004). What does it mean to involve consumers successfully in NHS research? A consensus study. Health Expect.

[58] Oxman AD, Lewin S, Lavis JN, Fretheim A (2009). SUPPORT tools for evidence-informed health policymaking (STP) 15: Engaging the public in evidence-informed policymaking. Health Res Policy Syst.

[59] Higgins JW (1999). Closer to home: The case for experiential participation in health reform. Can J Public Health.

[60] Kreis J, Puhan MA, Schünemann HJ, Dickersin K (2013). Consumer involvement in systematic reviews of comparative effectiveness research. Health Expect.

[61] Ruggiano N (2012). Consumer direction in long-term care policy: Overcoming barriers to promoting older adults’ opportunity for self-direction. J Gerontol Soc Work.

[62] Horowitz CR, Robinson M, Seifer S (2009). Community-based participatory research from the margin to the mainstream: Are researchers prepared?. Circulation.

[63] Wallen GR, Middleton KR, Miller-Davis C, Tataw-Ayuketah G, Todaro A, Rivera Goba M (2012). Patients’ and community leaders’ perceptions regarding conducting health behavior research in a diverse, urban clinic specializing in rheumatic diseases. Prog Community Health Partnersh.

[64] Andrews JO, Cox MJ, Newman SD, Gillenwater G, Warner G, Winkler JA (2013). Training partnership dyads for community-based participatory research: Strategies and lessons learned from the community engaged scholars program. Health Promot Pract.

[65] Gwede CK, Ashley AA, McGinnis K, Montiel-Ishino FA, Standifer M, Baldwin J (2013). Designing a community-based lay health advisor training curriculum to address cancer health disparities. Health Promot Pract.

[66] Stewart RJ, Caird J, Oliver K, Oliver S (2011). Patients’ and clinicians’ research priorities. Health Expect.

[67] Chiu CG, Mitchell TL, Fitch MI (2013). From patient to participant: Enhancing the validity and ethics of cancer research through participatory research. J Cancer Educ.

[68] Perkins DD (1995). Speaking truth to power: Empowerment ideology as social intervention and policy. Am J Community Psychol.

[69] Hewitt G, Draper AK, Ismail S (2013). Using participatory approaches with older people in a residential home in Guyana: Challenges and tensions. J Cross Cult Gerontol.

[70] Abelson J, Forest PG, Casebeer A, Mackean G (2004). Will it make a difference if I show up and share? A citizens’ perspective on improving public involvement processes for health system decision-making. J Health Serv Res Policy.

[71] Paterson C (2004). ‘Take small steps to go a long way’ consumer involvement in research into complementary and alternative therapies. Complement Ther Nurs Midwifery.

[72] Rhodes P, Nocon A, Wright J, Harrison S (2001). Involving patients in research: Setting up a service users’ advisory group. J Manag Med.

[73] Kowal E, Anderson I, Bailie R (2005). Moving beyond good intentions: Indigenous participation in aboriginal and Torres Strait Islander health research. Aust N Z J Public Health.

[74] Ross F, Donovan S, Brearley S, Victor C, Cottee M, Crowther P (2005). Involving older people in research: Methodological issues. Health Soc Care Community.

[75] Gargioni G (2010). Community healthcare programmes: Empowering people to improve access to prevention and care. J Med Pers.

[76] Huang CL, Wang HH (2005). Community health development: What is it?. Int Nurs Rev.

[77] Hempenius L, Slaets JPJ, Boelens MAM, Van Asselt DZB, de Bock GH, Wiggers T (2013). Inclusion of frail elderly patients in clinical trials: solutions to the problems. J Geriatr Oncol.

[78] Adams A, Miller-Korth N, Brown D (2004). Learning to work together: developing academic and community research partnerships. WMJ.

[79] Concannon TW, Meissner P, Grunbaum JA, McElwee N, Guise J, Santa J (2012). A new taxonomy for stakeholder engagement in patient-centered outcomes research. J Gen Intern Med.

[80] Couzos S, Lea T, Murray R, Culbong M (2005). ‘We are not just participants-- we are in charge’: The NACCHO ear trial and the process for aboriginal community-controlled health research. Ethn Health.

[81] Kenny A, Hyett N, Sawtell J, Dickson-Swift V, Farmer J, O'Meara P (2013). Community participation in rural health: A scoping review. BMC Health Serv Res.

[82] Sinding C, Miller P, Hudak P, Keller-Olaman S, Sussman J. Of time and troubles: Patient involvement and the production of health care disparities. Health. 2012;16(4):400–17. 10.1177/1363459311416833 21856716

[83] Molster C, Potts A, McNamara B, Youngs L, Maxwell S, Dawkins H (2013). Informing public health policy through deliberative public engagement: Perceived impact on participants and citizen-government relations. Genet Test Mol Biomarkers.

[84] Butt G, Markle-Reid M, Browne G (2008). Interprofessional partnerships in chronic illness care: a conceptual model for measuring partnership effectiveness. Int J Integr Care.

[85] Lüdecke D (2014). Patient centredness in integrated care: results of a qualitative study based on a systems theoretical framework. Int J Integr Care.

[86] Ferrer L, Goodwin N (2014). What are the principles that underpin integrated care?. Int J Integr Care.

[87] Carman KL, Dardess P, Maurer M, Sofaer S, Adams K, Bechtel C, Sweeney J. Patient and family engagement: a framework for understanding the elements and developing interventions and policies. Health Aff (Millwood). 2013. doi: 10.1377/hlthaff.2012.1133. 10.1377/hlthaff.2012.1133 23381514

[88] Supple D, Roberts A, Hudson V, Masefield S, Fitch N, Rahmen M, et al. From tokenism to meaningful engagement: best practises in patient involvement in an EU project. Res Involv Engage. 2015;1(5). doi: 10.1186/s40900-015-0004-9. 10.1186/s40900-015-0004-9 PMC5598090 29062494

[89] Nauta AP, von Grumbkow J (2001). Factors predicting trust between GPs and Ops. Int J Integr Care.

[90] Rousseau DM, Sitkin SB, Burt R, Camerer C (1998). Not so different after all: a cross discipline view of trust. Acad Manage Rev.

[91] Glenny C, Stolee P, Sheiban L, Jaglal S (2013). Communicating during care transitions for older hip fracture patients: family caregiver and health care provider’s perspectives. Int J Integr Care.

[92] Pirnejad H, Bal R, Stoop A, Berg M (2007). Inter-organisational communication networks in healthcare: centralised versus decentralised approaches. Int J Integr Care.

[93] Juhnke C, Muhlbacher A (2013). Patient-centredness in integrated healthcare delivery systems - needs, expectations and priorities for organised healthcare systems. Int J Integr Care.

